# Effects of a Multi-Component Mycotoxin-Detoxifying Agent on Oxidative Stress, Health and Performance of Sows

**DOI:** 10.3390/toxins15090580

**Published:** 2023-09-19

**Authors:** Vasileios G. Papatsiros, Christos Eliopoulos, Nikolaos Voulgarakis, Dimitrios Arapoglou, Insaf Riahi, Meritxell Sadurní, Georgios I. Papakonstantinou

**Affiliations:** 1 Clinic of Medicine, Faculty of Veterinary Medicine, University of Thessaly, 43100 Karditsa, Greece; nvoulgarakis@uth.gr; 2 Institute of Technology of Agricultural Products, Hellenic Agricultural Organization-Demeter (HAO-Demeter), 14123 Athens, Greece; chris_eliopoulos@hotmail.com (C.E.); dimarap@yahoo.com (D.A.); 3 BIŌNTE Animal Nutrition, 43204 Reus, Spain; insaf.riahi@bionte.com (I.R.); meritxell.sadurni@bionte.com (M.S.)

**Keywords:** mycotoxin, detoxifying agent, curcumin, silymarin, yeast, TBARS, CARBS, TAC, sow, pig

## Abstract

This in vivo study aimed to investigate the effects of a multi-component mycotoxin-detoxifying agent, containing clays (bentonite, sepiolite), phytogenic feed additives (curcumin, silymarin) and postbiotics (yeast cell wall, hydrolyzed yeast) on the antioxidant capacity, health and reproductive performance of pregnant and lactating sows challenged by mycotoxins. Eighty (80) primiparous sows (mean age 366 ± 3 days) per each of the two trial farms were divided into two groups in each farm: a) T1 (control group): 40 sows received the contaminated feed and b) T2 group (experimental group): 40 sows received the contaminated feed plus the mycotoxin-detoxifying agent, one month before farrowing until the end of the lactation period. Thiobarbituric acid reactive substances (TBARS), protein carbonyls (CARBS) and total antioxidant capacity (TAC) were evaluated as biomarkers of oxidative stress. Clinical and reproductive parameters were recorded. Our results indicate that the administration of a multi-component mycotoxin-detoxifying agent’s administration in sow feed has beneficial effects on oxidative stress biomarkers and can improve sows’ health and performance.

## 1. Introduction

Mycotoxins are secondary metabolites produced by fungi (moulds), and they are widely distributed in food and animal feed worldwide. Aspergillus and Fusarium species are the most important plant pathogens that infect food crops and produce mycotoxins in animal feed [1,2,3]. Mycotoxins, even when detected at low levels, are toxic to human and animal health [4,5]. The most common mycotoxins found in animal feed are aflatoxins (AFs, aflatoxin B1-AFB1, aflatoxin B2-AFB2, aflatoxin G1-AFG1, aflatoxin G2-AFG2); ochratoxin A (OTA); trichothecenes: deoxynivalenol (DON), T2 toxin (T-2) and HT 2 toxin (HT-2); fumonisins (FUBs, fumonisin B1-FUB1, fumonisin B2 FUB-2) and zearalenone (ZEN) [6].

Mycotoxins represent a critical risk factor for pig health since they extensively contaminate cereals, which are the most common ingredient in pig feed [7]. Ingestion of mycotoxins, even at low levels, can lead to acute, subacute or chronic disease (mycotoxicosis) [2,8,9], causing significant economic losses. In particular, dietary ingestion of mycotoxins can cause severe damage to the immune [10], reproductive [11] and respiratory systems [12], as well as to various organs such as the intestine [13,14], kidney [15] and liver [16,17]. In sows, mycotoxins cause (a) decreased feed intake, (b) problems in piglet development [18], (c) liver damage [19], (d) gastrointestinal tract dysfunction (GI) and oxidative stress [20], (e) various reproductive and infant system dysfunctions [21,22] and (d) offspring health problems (e.g., swollen vulva in suckling piglets, increased indicators of oxidative stress) [23,24,25]. In addition, they are considered a crucial risk factor for postpartum dysgalactia syndrome (PDS) [26].

The most common approach to prevent and control mycotoxins is the administration of mycotoxin detoxifiers in the form of feed additives [27,28,29,30,31]. Nowadays, their use is common practice in the swine industry to reduce mycotoxin toxicity in contaminated feed ingredients (e.g., grains) and provide safer raw materials for pig feed production [32,33,34,35]. Commercial mycotoxin detoxification agents can be divided into two types: (a) adsorbents (mycotoxin binders) and (b) biotransformation agents (mycotoxin modifiers), which are designed to prevent the uptake of mycotoxins via GI and into the bloodstream [36]. The mode of action of the first agent is the adsorption of mycotoxins in GI, which promotes the excretion of mycotoxin-binder complexes in feces and prevents the uptake of mycotoxins. In contrast, the action of biotransformants is different and aims to transform mycotoxins by using microorganisms/enzymes into metabolites with non- or less-toxic properties [37,38,39,40]. The complex passes through the GI, and its excretion via feces results in the reduced uptake of mycotoxins by various organs. Mycotoxin binders or mycotoxin modifiers often contain multiple components (e.g., clay, yeast products, phytogenics, herbs) to improve absorbability, immune function, and gut health and reduce oxidative stress [41] or provide essential nucleotide sources (e.g., dietary fiber, nucleotides) to improve their detoxification properties [42,43]. Furthermore, several studies have shown that feeding yeast products to sows during lactation has a positive effect on the growth performance of their piglets by increasing colostrum yield and feed intake and improving piglet health and immunity [44,45,46,47,48]. In addition, yeast products have been suggested to prevent the negative effects of feed contaminated with several mycotoxins on weaning piglets [49].

Moreover, silymarin has been characterized by hepatoprotective, antioxidant and anti-inflammatory properties [50,51,52,53,54]. It is extracted from milk thistle (*Silybum marianum*) and consists of four main component isomers (silybin, isosilybin, silydianin and silychristin) [55]. Several studies have reported beneficial effects of silymarin supplementation in pregnant and lactating sows, such as anti-inflammatory effects (also associated with modulation of gut microbiota), increased antioxidant capacity of the sow and decreased oxidative stress, improved milk yield, reproductive and litter performance (e.g., increased sow colostrum/milk yield and feed intake, decreased body weight loss during lactation and heavier suckling and weaned piglets) [56,57,58,59,60].

This in vivo study aimed to assess the effects of a multi-component mycotoxin detoxifier on antioxidant capacity, health and reproductive performance of pregnant and lactating sows contaminated with mycotoxins.

## 2. Results

### 2.1. Mycotoxins Detection and Quantification in the Feed of Trial Farms

Laboratory testing for mycotoxins in the feed samples from both farms resulted in the detection of several mycotoxins, as shown in Table 1. The feed from experimental farm 1 was contaminated with FUB1, FUB2, ZEN and T2 toxin several times. On the other hand, the feed from experimental farm 2 was contaminated several times with FUB1, at levels higher than those prescribed in Europe, as well as with FUB2 and AFB1.

### 2.2. Oxidative Stress Biomarkers

Table 2 shows the TBARS (μmol/L), CARBS (nmol/L) and TAC (mmol/L) values in plasma (μmol/L) between groups per experimental condition. Statistically significant differences between groups T1 and T2 were observed for all biomarker levels in the plasma of sows (Table 2). TBARS and CARBS levels were significantly higher in sows from experimental farm 2 compared with experimental farm 1, whereas TAC levels were lower.

### 2.3. Clinical and Performance Parameters

The evaluation of clinical parameters between groups in the different experimental farms is shown in Table 3. Statistically significant differences were found between the control and experimental groups in both experimental farms (Table 3).

The evaluation of bedding characteristics between groups in the different experimental farms is shown in Table 4. Statistically significant differences were found between the control and experimental groups in both experimental farms (Table 4), indicating a statistical increase in the mean number of piglets born alive and weaned and a significant decrease in the mean number of piglets and mothers born dead in the T2 group in both experimental farms.

## 3. Discussion

This study aimed to evaluate the effects of a multi-component mycotoxin detoxifier on antioxidant capacity, health and reproductive performance of pregnant and lactating sows contaminated with mycotoxins. In general, feed contaminated with multiple mycotoxins is a risk factor for livestock [62]. Pigs show high susceptibility to feeds co-contaminated with multiple mycotoxins [63,64,65]. In our study, we also found that multiple mycotoxin contamination of sow diets, even with low-to-moderate levels of mycotoxins, had negative effects on sow performance parameters and litter characteristics. Our results are in agreement with previous studies that reported poor reproductive and clinical performance of sows due to AFB1, FUB1, ZEN and T2 contamination [66,67,68]. Zinedine et al. (2007) [68], like our results, reported severe reproductive problems in sows exposed to contaminated feed with low concentrations of ZEN. Nevertheless, in an SPF (specific pathogen-free) herd, naturally, moderately mycotoxin-contaminated feed was shown to have limited effects on sow health and production during late gestation and lactation [69]. The European Union has announced maximum levels for mycotoxins in feed [70,71,72,73,74]. Avoiding the use of contaminated grain is the ideal strategy to prevent the negative effects of mycotoxins, but in practice, the use of contaminated feed is very difficult to avoid. Moreover, the increasing frequency of contamination of feeds with several mycotoxins indicates that mycotoxin detoxification agents are becoming more necessary as an efficient strategy [70].

Mycotoxins have negative effects on the antioxidant system of pigs by causing an increase in the toxic lipid peroxidation byproduct MDA and inhibiting the activity of antioxidant defense mechanisms. The increased number of free radicals and inhibition of the antioxidant system cause damage to DNA, proteins and lipids [75]. However, the extent of oxidative stress depends on several factors, including co-contamination with multiple mycotoxins and their detected concentrations or synergistic effects, the duration of mycotoxin exposure and the age and production stage of the exposed animals [76]. In our study, increased levels of the oxidative stress biomarkers TBARS and CARBS were detected in the plasma of sows from experimental farm 1 that had received feed co-contaminated with multiple mycotoxins, confirming the results of the above studies. Moreover, TBARS and CARBS levels were significantly higher in sows from experimental farm 2, which received feed co-contaminated with overall higher FUB levels (6489.5 ppb), than in experimental farm 1 (3468.0 ppb), while TAC levels were lower. Several researchers have indicated the mechanisms by which Afs, ZEN, T2 toxins and especially FUBs have negative effects on the antioxidant system of pigs. In particular, FUBs can stimulate the cells or tissues of animals to produce active oxygen and inhibit the activities of antioxidant enzymes [77,78]. However, a shortcoming of our study is that blood sampling was performed at a single time point during the sow feeding period and all experimental groups were continuously exposed to mycotoxins. Therefore, it was not possible to study the effect of mycotoxins compared to sows fed diets not contaminated with mycotoxins.

Curcumin and silymarin are phytogenics with known antioxidant properties. In the present study, the mycotoxin detoxifier tested contained plant extracts of curcumin and silymarin. Previous in vitro studies reported the antioxidant properties of curcumin in cells exposed to the mycotoxin ZEN. Similarly, other in vitro studies reported the antioxidant effects of curcumin and silymarin on mycotoxins OTA, FUB1 and DON [79]. However, our study is the first in vivo study to investigate the antioxidant capacity of curcumin as a mycotoxin detoxifying agent in sows under field conditions with multi-mycotoxin contamination. The combination of multiple mycotoxins in naturally contaminated feeds at low concentrations can have synergistic and negative additive effects [80,81], even when levels are below regulatory guidelines [70]. Previous in vivo studies reported positive hepatoprotective and antioxidant effects of silymarin against mycotoxins AFB1 and OTA in poultry and rodents [82,83]. Our study confirmed these previous reports, including in vivo experiments in sows under field conditions with multiple contamination of mycotoxins, as a significant decrease in oxidative stress biomarkers (TBARS, CARBS) and an increase in antioxidant capacity (TAC) indicators were observed. In our study, a significant improvement in sow bedding characteristics and health was observed, which was attributed to the possible detoxifying effect of the beneficial effects of phytogenics and postbiotics. The tested phytogenics (curcumin and silymarin) can effectively improve antioxidant capacity [52,53,54,55,84,85,86,87]. In particular, several studies have shown that they can neutralize ROS by increasing the level of antioxidant enzymes in serum, thus playing an important role in the defense against oxidative stress caused by FUBS, Afs, ZEN and T2 toxins as the first line of defense. Thus, curcumin and silymarin can be used as antioxidant feed additives to repair damage that may be caused by these mycotoxins [88]. Our results confirmed the findings of previous studies using silymarin as a dietary supplement in pregnant and lactating sows, which demonstrated its anti-inflammatory effects (increased antioxidant capacity and decreased oxidative stress) and positive effects on reproductive parameters and litter performance [56,57,58,59,60]. However, our study is the first field study with the use of curcumin in pregnant and lactating sows in diets contaminated with multiple mycotoxins, as previous studies have limitedly investigated the effects of curcumin on IUGR piglets [85,86,87,89]. In addition, we investigated the effects of silymarin under field conditions with multi-mycotoxin contamination.

The mycotoxin detoxifier tested in our study also contains postbiotics (yeast cell walls and hydrolyzed yeast) and, according to our results, may contribute to lowering the level of oxidative stress biomarkers (TBARS, CARBS) in the plasma of sows and improving sow health and performance. Vila-Donat et al. (2018) [90] pointed out that yeasts can transform toxins into non-toxic or at least less-toxic products, while some of them can suppress the development of filamentous fungi. The use of yeasts in various technological processes may have a direct inhibitory effect on the synthesis of toxins by certain fungi, while several species could accumulate mycotoxins from agricultural products, thereby successfully detoxifying them. Postbiotics have been also reported to prevent the negative effects of feed contaminated with several mycotoxins on weanling health and growth performance [41]. Several studies have shown that feeding yeast products to sows during the lactation period has a positive effect on the growth performance of their piglets due to increased colostrum yield and feed intake, improvement of piglet immunity, and increased exposure of piglets to beneficial microorganisms via sow feces [45,46,47,48,91]. In addition, yeast products could protect against mycotoxins due to their ß-D-glucan composition (increasing absorption and decreasing the negative effects of mycotoxins on various organs), thus improving pig growth and health [44,47,91,92,93,94,95,96,97]. In our study, administration of the tested mycotoxin detoxifier containing yeast products as postbiotics could contribute to decreasing the level of oxidative stress biomarkers and improve the reproductive and litter parameters of sows (e.g., decrease in dead-born and increase in live-born piglets), which is probably due to the positive effects of phytogenics and postbiotics on sow health and reproduction according to previous studies [45,46,47,48,84]. Mycotoxins are also associated as the main risk factor with clinical cases of PDS under field conditions [26]. In our study, the clinical parameters associated with the clinical performance of PDS (pyrexia, udder formation, pain, etc.) were significantly reduced in the groups receiving the tested mycotoxin detoxifier with phytogenics and postbiotics. PDS is important to the modern swine industry because it affects sow longevity, resulting in economic losses [26,98,99]. Based on our results, the use of mycotoxin detoxifiers in pregnant and lactating sows could reduce the clinical cases of PDS or mitigate the consequences of PDS, thus significantly reducing economic losses.

## 4. Conclusions

This study showed that the administration of a multi-component mycotoxin detoxifier in sow diets containing clays (bentonite, sepiolite), phytogenics (curcumin, silymarin), and postbiotics (yeast cell wall, hydrolyzed yeast) had beneficial effects on biomarkers of oxidative stress and improved sow health and performance under various mycotoxin loads. The animals in our field study were exposed to multiple mycotoxins, and our results provide a basis for future in vivo studies to investigate the detailed degradation mechanisms of the antioxidant effects of the multi-component mycotoxin detoxifier tested, which contains more compounds than typical toxin binders (such as plant extracts of curcumin and silymarin, yeast cell walls, etc.). Further studies are needed in the future to investigate the losses due to feed contaminated with multiple mycotoxins and the benefits of routine administration of mycotoxin detoxifiers at different ages in commercial swine herds.

## 5. Materials and Methods

### 5.1. Trial Farms: Animals and Diets

The present study was conducted on two commercial farrow-to-finish farms in central Greece (Thessaly) from January 2023 to May 2023. Farm 1 had a capacity of 600 sows (Large White × Landrace, DanBred) and Farm 2 had a capacity of 560 sows (Large White × Landrace).

All gilts/sows on both farms were individually ear-tagged and housed in a separate mating building where they were artificially inseminated with semen cans from Duroc boars at the same boar stud. On the day of weaning, sows were moved to the mating building and housed in individual cages with slatted floors and separate feeding stalls until artificial insemination. The inseminated sows remained in individual cages until the 25th to 30th day of gestation when they were moved to group housing until one week before the expected farrowing day. The weaning age of piglets was 25–28 days, and weaned piglets were transferred weekly to the flat deck unit in pens with groups of 25. A one-week farrowing series and a 25- to 28-day lactation period were conducted on the experimental farms, so approximately 40–50 sows were grouped per series.

Sows on the experimental farms were housed in farrowing crates, and litters were usually standardized within the first 24 h after birth. During the last week of gestation, sows were moved to the farrowing room. Pregnancy and lactation diets for the sows are shown in Table 5, including the home-mixed corn/barley/wheat/soybean-based diet, according to the recommendations of the National Research Council [100]. From one week to 3 days before the expected farrowing day, the feed was given twice daily. The diet of the suckling piglets in the experimental farms, apart from the mother’s milk, included a milk replacer from the first day after farrowing. From 7 days of age, piglets were given a commercial creep feed daily until weaning. Farrowing pens were equipped with nipple drinkers and separate removable drinkers for the sows and piglets in each farrowing pen. All animals had free access to fresh water throughout the lactation period. In addition, the barns in the experimental farms were equipped with a fully automated feeding system for the weaners and a climate control system for temperature and humidity in the farrowing and weaning barns. Sows were vaccinated against Aujeszky’s disease, erysipelas, parvovirus, atrophic rhinitis, porcine reproductive and respiratory syndrome, porcine circovirus, swine influenza, colibacillosis and clostridia. A single ivermectin injection was administered two weeks prior to farrowing for the antiparasitic control sows.

### 5.2. Laboratory Examinations for Mycotoxins in Feed

Prior to the start of the trial on the experimental farms, samples (500 g) of the sows’ finishing feed were analyzed and quantified for mycotoxins on both farms. The tests were performed in APSA LAB (a company of the Pintaluba Group, Andrés Pintaluba S.A., Reus, Spain) according to the following methodology. In vitro mycotoxin extraction from feed samples was performed by weighing 5 g of the sample into a centrifuge tube. Previously, the feed samples were ground to a fine powder using a laboratory mill with a sieve. Then, 20 mL of extraction solution was added to the tube (80% acetonitrile–water solution with 0.1% formic acid), and it was shaken for 1 h. The sample was then centrifuged at 3500 rpm and 20 °C for 5 min. The liquid fraction was transferred to another tube and stored for later use. Another 20 mL of extraction solution was added to the remaining solid, it was shaken for another 30 min and then centrifuged at 3500 rpm and 20 °C for 5 min. The liquid phase was transferred to the same tube as the one previously reserved. The approximately 40 mL of extraction liquid was centrifuged at 3500 rpm and 20 °C for 5 min. Then, 1 mL was transferred to an Eppendorf tube and centrifuged at 12,500 rpm for 5 min. Finally, 80 µL of the solution was transferred to a high-pressure liquid chromatography (HPLC) vial and 20 µL of internal standard was added for instrument injection according to the method described by Stroka et al. (2000) [101]. The calibration curve was prepared using commercial standard solutions. In addition, to correct for matrix effect, the internal standard was previously prepared using a 28% acetonitrile–water solution. These mycotoxin standards were labelled with C13 isotopes. Quantification of eleven mycotoxins (AFB1, AFB2, AFG1, AFG2, FUB1, FUB2, OTA, ZEN, DON, T-2 and HT -2) was performed using a HPLC-MS technique. A Zorbax RRHD Eclipse Plus C18 (2.1 × 100, 1.8 µm) column was used to separate the analytes. The flow rate was 0.3 mL/min, and the mobile phase consisted of a 0.1% formic acid-5 mM ammonium formate water solution (A) and a 0.1% formic acid-5 mM ammonium formate methanol solution (B) in a gradient as follows: Initial conditions were 30% for 0.5 min, conditions changed to 100% of B and were maintained for 9 min. Finally, we returned to the initial conditions after 0.1 min. The total analysis time was 16.5 min. The injection volume was 3 µL and the column was maintained at 30 °C.

### 5.3. Experimental Material

In this field trial, an innovative agent for detoxification of mycotoxins (BIŌNTE^®^ QUIMITŌX^®^ PLUS, BIŌNTE NUTRITION S.L., Reus-Tarragona, Spain) was tested. This remedy contains bentonite and sepiolite, phytogenics (natural extracts of silymarin and curcumin) and a combination of selected yeast extracts (yeast cell wall and hydrolyzed yeast).

### 5.4. Study Design

#### 5.4.1. Study 1

Eighty (80) primiparous sows (mean age 366 ± 3 days) were selected from a single lot 15 days before the expected farrowing date in experimental farm 1 because they had no previous health history and similar body weights (206.8 ± 5.7 kg). The primiparous sows were divided into two groups: (a) T1 (control group): 40 sows received the contaminated feed (see Table 1) and (b) T2 group (experimental group): 40 sows received the contaminated feed (see Table 1) plus 1.5 kg of a multi-component mycotoxin detoxifier (BIŌNTE^®^ QUIMITŌX^®^ PLUS) one month before farrowing until the end of the lactation period.

#### 5.4.2. Study 2

Eighty (80) primiparous sows (mean age 363 ± 4 days) were selected from a single lot 15 days prior to the expected farrowing date in experimental farm 2 because they had no previous health history and similar body weights (204.5 ± 6.3 kg). The primiparous sows were divided into two groups: (a) T1 (control group): 40 sows received the contaminated diet (see Table 1) and (b) T2 group (experimental group): 40 sows were fed the contaminated diet (see Table 1) plus 2.5 kg of a multi-component mycotoxin detoxifier (BIŌNTE^®^ QUIMITŌX^®^ PLUS) one month before farrowing and 1 kg of the same mycotoxin detoxifier during the lactation period.

### 5.5. Blood Sampling

Blood samples were collected via jugular vein puncture from 20 sows per group in both experimental farms, fixed with a snare during the first 24 h after farrowing, using BD Vacutainer^®^ plasma tubes (Becton Dickinson, Franklin Lakes, NJ, USA,) with EDTA as anticoagulant. Plasma was collected via centrifugation of the samples at 12,000× *g* for 10 min at 4 °C. The resulting plasma was transferred to 1.5-mL microcentrifuge tubes and stored frozen at −80 °C until analysis.

### 5.6. Laboratory Examinations for Oxidative Stress Biomarkers

Thiobarbituric acid reactive substances (TBARS) and protein carbonyls (CARBS) were determined in plasma as biomarkers of oxidative stress according to Gerasopoulos et al. (2015) [102]. A modified assay by Keles et al. (2001) [103] was used for TBARS determination. TBARS concentration was calculated based on the molar extinction coefficient of malondialdehyde (MDA). CARBS content was determined according to Patsoukis et al. (2004) [104] and was based on the molar extinction coefficient of 2,4-dinitrophenylhydrazine. In addition, the determination of total antioxidant capacity (TAC) in plasma was performed according to the method of Janaszewska and Bartosz (2002) [105].

### 5.7. Clinical Examination and Records

Clinical examination of sows participating in Study-1 and Study-2 was performed by recording (a) rectal temperature 24 h after farrowing, using a scoring system (0 = normal, up to 40 °C, 1 => 41 °C, 2 => 42 °C, (b) anorexia and vulvar discharge, (c) litter characteristics (average total number of live-born, stillborn, mummified, and weaned piglets), (d) general clinical signs, and (e) findings from examination of the udder (left and right sides) to find sows affected by postpartum dysgalactia syndrome (PDS). In particular, individual clinical examination of the mammary glands was performed using a modified scoring system according to Spiegel (2016) [106] and Rosengart et al. (2021) [107] as follows: (a) shape/regression (0 = in lactation, 1 = poor shape/regression, 2 = not shaped/no milk production), (b) skin color (0 = normal skin color, 1 = moderately red, 2 = severely red), (c) consistency (0 = loose, 1 = elastic, 2 = firm), (d) nodes (0 = absent, 1 = present in skin/subcutis, 2 = present in breast parenchyma), (e) pain (0 = absent, 1 = mild, 2 = severe). For scratches and teat injuries, only yes and no were distinguished. The evaluation of pain was based on the grade of findings after the palpation of the udder, including frequencies of posture changes and durations of postural behavior (e.g., aggression) [108] and the facial expression (tension above the eyes, snout angle and neck tension) [109]. For the other characteristics, three gradations were made.

### 5.8. Statistical Analysis

All analyses were performed in triplicate. The results were expressed as means ± standard deviation (±S.D.). Data normality was assessed using the Kolmogorov–Smirnov and Shapiro–Wilk tests. The differences between two groups were assessed using independent and paired samples *t*-tests. Statistical significance was accepted at a confidence level of 95% (*p* ≤ 0.05).

## Figures and Tables

**Table 1:** Detected mycotoxins in the feed of trial farms.

Detected Mycotoxin (ppb)	Farm-1	Farm-2	Maximum Level (ppb) According to EU Regulation *
Total FUBs	3468.0	6489.5	(FUB1 + FUB2) 5000
FUB1	2767.5	5109.4	
FUB2	700.5	1380.1	
ZEN	14.6	-	100
AFB1	-	5.1	20
T2 toxin	4.2	-	250
Clinical guide to mycotoxins affecting sows [61]			
Mycotoxin	Dietary level (ppb or ppm) 1 ppm = 1000 ppb	Effects	
AFs	>2000 ppb	acute hepatosis, high mortality rate	
	400 to 800 ppb	slow-growing suckling pigs due to aflatoxin in milk	
ZEN	1–3 ppm	vulvovaginitis, prolapses of rectum and vagina	
	3–10 ppm	anestrus, pseudopregnancy	
	>30 ppm	early embryo loss	
T2 toxin	3 ppm	low feed intake	
	10 ppm	low feed intake; oral/dermal irritation; immune-suppression	
	20 ppm	Anorexia, vomiting	
FU Bs	<20 ppm	No signs	
	50 to 100 ppm	decreased feed intake, decreased weight gain, hepatosis	
	>100 ppm	Acute pulmonary edema, hepatosis, and death	

* European Commission (EC) 2006/576/EC, and 2002/32/EC.

**Table 2:** Levels (μmol/L, nmol/L, mmol/L) of oxidative stress biomarkers (mean ± sd) of sows in trial farms.

Parameters	Farm-1			Farm-2		
	Groups			Groups		
	T1	T2	*p* Value	T1	T2	*p* Value
TBARS	5.61 ± 0.96 ^a^	3.83 ± 0.70 ^b^	0.009	7.78 ± 0.67 ^a^	4.97 ± 1.04 ^b^	<0.001
CARB	0.73 ± 0.07 ^a^	0.47 ± 0.11 ^b^	<0.001	0.79 ± 0.07 ^a^	0.51 ± 0.06 ^b^	<0.001
TAC	0.46 ± 0.01 ^b^	0.80 ± 0.02 ^a^	<0.001	0.31 ± 0.03 ^b^	0.72 ± 0.02 ^a^	0.007

Different superscripts in the same row per farm indicate statistical significance (*p* ≤ 0.05).

**Table 3:** Clinical and performance parameters (mean ± sd) of sows in trial farms.

Parameters	Farm-1			Farm-2		
	T1 Group (*n* = 40)	T2 Group (*n* = 40)	*p* Value	T1 Group (*n* = 40)	T2 Group (*n* = 40)	*p* Value
Temperature −24 h (0 = normal, up to 40 °C, 1 => 41 °C 2 => 42 °C)	1.25 ± 0.68 ^a^	0.23 ± 0.42 ^b^	<0.001	1.27 ± 0.71 ^a^	0.29 ± 0.56 ^b^	<0.001
Mammary glands						
Shaping/Regression (0 = in lactation, 1 = poor shape/regression, 2 = not shaped/no milk production)	1.25 ± 0.68 ^a^	0.27 ± 0.45 ^b^	<0.001	1.31 ± 0.82 ^a^	0.27 ± 0.45 ^b^	<0.001
Skin color (0 = normal skin color, 1 = moderately red, 2 = severely red)	1.25 ± 0.71 ^a^	0.35 ± 0.48 ^b^	<0.001	1.33 ± 0.79 ^a^	0.47 ± 0.56 ^b^	<0.001
Consistency (0 = loose, 1 = elastic, 2 = firm)	1.10 ± 0.38 ^a^	0.30 ± 0.48 ^b^	<0.001	1.32 ± 0.63 ^a^	0.37 ± 0.67 ^b^	<0.001
Nodes (0 = absent, 1 = present in skin/subcutis, 2 = present in breast parenchyma)	0.67 ± 0.47 ^a^	0.28 ± 0.45 ^b^	<0.001	0.72 ± 0.56 ^a^	0.33 ± 0.52 ^b^	<0.001
Pain (0 = absent,1 = mild, 2 = severe)	1.33 ± 0.73 ^a^	0.35 ± 0.48 ^b^	<0.001	1.46 ± 0.95 ^a^	0.55 ± 0.64 ^b^	<0.001

Different superscripts in the same row per each trial farm indicate statistical significance (*p* ≤ 0.05).

**Table 4:** Litter characteristics (mean ± sd) of sows in trial farms.

Parameters	Farm-1			Farm-2		
	T1 Group (*n* = 40)	T2 Group (*n* = 40)	*p* Value	T1 Group (*n* = 40)	T2 Group (*n* = 40)	*p* Value
Total Born	15.78 ± 1.58 ^a^	15.75 ± 1.49 ^a^	0.926	15.62 ± 1.24 ^a^	15.65 ± 1.32 ^a^	0.875
Dead Born	1.38± 0.74 ^a^	0.78 ± 0.48 ^b^	<0.001	1.76± 0.89 ^a^	0.87 ± 0.52 ^b^	<0.001
Live Born	13.08 ± 0.99 ^a^	14.43 ± 1.19 ^b^	<0.001	12.28 ± 0.92 ^a^	14.38 ± 1.19 ^b^	<0.001
Mummies	1.35 ± 0.69 ^a^	0.55 ± 0.50 ^b^	<0.001	1.65 ± 0.78 ^a^	0.42 ± 0.39 ^b^	<0.001
Fostering taking	0.48 ± 0.82 ^a^	0.18 ± 0.45 ^b^	0.045	0.32 ± 0.82 ^a^	0.16 ± 0.34 ^b^	0.055
Fostering giving	1.40 ± 0.98 ^a^	0.78 ± 0.62 ^b^	0.002	1.54 ± 0.97 ^a^	0.62 ± 0.53 ^b^	0.003
Weaned	10.93 ± 1.89 ^a^	13.28 ± 0.72 ^b^	<0.001	10.08 ± 1.65 ^a^	13.56 ± 0.87 ^b^	<0.001

Different superscripts in the same row per each trial farm indicate statistical significance (*p* ≤ 0.05).

**Table 5:** Feeding schedule, diet composition and nutrient content of gestation feed (GF) and lactation feed (LF) of sows’ diet in the trial farms.

Composition of Ingredients (kg)	Trial Farm-1		Trial Farm-2	
	GF	LF	GF	LF
Corn	300.0	345.0	300.0	328.0
Barley	280.0	200.0	470.0	400.0
Wheat bran	235.0	190.0	-	-
Soybean meal (46% crude protein)	120.0	170.0	72.5	148
Sunflower (28% crude protein)	-	-	75.0	25.0
Sugar beet	-	-	50	25.0
Soybean oil	10.0	20.0	-	18.0
Protein concentrate (68% crude protein)	14.0	24.0		
Fish meal	-	-		25.0
Vitamins/minerals premix	30.0	40.0	30.0	30.0
Inactive dried yeast	5.0	5.0	-	-
Mycotoxin binder	1.5	1.5	2.5	1.0
Dietary cellulose powder	4.5	4.5	-	-
Total	1000	1000	1000	1000
Analyzed nutrient compositions (%)	GF	LF	GF	LF
Crude protein	16.50	18.40	13.63	16.92
Crude fat	3.70	4.65	2.14	4.06
Crude fiber	5.40	4.70	6.32	4.53
Lysine	0.80	0.98	0.6	1.04
Methionine	0.29	0.33	0.20	0.35
Methionine + Cystine	0.60	0.63	0.80	0.60
Calcium	0.65	0.86	0.95	0.98
Total phosphorus/available phosphorus	0.76/0.40	0.78/0.46	0.65/0.35	0.67/0.38
Sodium	0.24	0.24	0.24	0.24

## Data Availability

All data generated for this study are presented within the manuscript.

## Abbreviations

AFB1	Aflatoxin B1
AFB2	Aflatoxin B2
AFG1	Aflatoxin G1
AFG2	Aflatoxin G2
Affs	Aflatoxins
AI	Artificial Insemination
CARBS	Protein Carbonyls
DON	deoxynivalenol
FUB1	Fumonisin B1
FUB2	Fumonisin B2
FUBsS	Fumonisins
GI	Gastrointestinal Tract
HPLC	High-Pressure Liquid Chromatography
HT2	Trichothecene Trichothecene Toxin HT2 toxin
IUGR	Intrauterine Growth Restriction
MDA	Malondialdehyde
OTA	Ochratoxin A
PDS	Postpartum Dysgalactia Syndrome
ROS	Reactive Oxygen Species
SD	Standard Deviation
SPF	Specific Pathogen-Free
T2	Trichothecene Toxin T2 toxin
TAC	Total Antioxidant Capacity
TBARS	Thiobarbituric acid reactive substances
ZEN	Zearalenone
